# Normotensive offspring of hypertensive Nigerians have increased Left ventricular mass and abnormal geometric patterns

**Published:** 2012-01-14

**Authors:** Philip Kolo, Emmanuel Sanya, James Ogunmodede, Ayodele Omotoso, Ayodele Soladoye

**Affiliations:** 1 Department of Medicine, University of Ilorin Teaching Hospital, P.M.B. 1459, Ilorin, Nigeria; 2 Department of Physiology, University of Ilorin, P.M.B. 1515, Ilorin, Nigeria

**Keywords:** Left ventricular mass, left ventricular geometry, echocardiogram, hypertension, hypertrophy

## Abstract

**Background:**

Reports have shown that normotensive offspring of hypertensive parents (OHP) are at increased risk of developing systemic hypertension (SH) and adverse cardiovascular events later in life. The pathological antecedents of this are thought to be alterations in the structure and function of left ventricle. Therefore, the present study aimed at determining left ventricular mass and geometry in OHP and compared with offspring without parental hypertension.

**Methods:**

Sixty-five OHP aged 15-25 years with 65-age and sex-matched offspring of normotensive parents (ONP) were studied for early makers of hypertensive cardiovascular disease. Those with heart murmurs, structural heart diseases and blood pressure ≥ 140/90 mmHg were excluded. Electrocardiography (ECG) and echocardiogram were done in standard positions.

**Results:**

Mean left ventricular posterior wall thickness, left ventricular mass, left ventricular mass index (LVMI) and relative wall thickness (RWT) were significantly higher in the subjects than controls (p=0.001, 0.046, 0.03 and 0.004 respectively). LVMI correlated positively with systolic and diastolic blood pressure, waist circumference (WC), ECG voltage, and posterior wall diastolic dimension. Waist circumference was an independent predictor of LVMI in OHP.

**Conclusion:**

We concluded that normotensive OHP have alterations in left ventricular mass and structure; and should be considered as a special group that needs early dietary and lifestyle adjustments to prevent future cardiovascular events.

## Background

Current approach to the management of systemic hypertension (SH) is early diagnosis and identification of individuals at risk through detection of early makers of the disease. The Writing Group of American Society of Hypertension considers left ventricular hypertrophy (LVH) as one of the early makers of hypertensive cardiovascular disease [1]. The presence of LVH in patients with SH is a strong and independent risk factor for future cardiovascular morbidity and mortality [2–3]. It is well known that normotensive OHP are at increased risk of developing SH later in life when compared with ONP [4]. However, previous studies particularly among Caucasians on cardiovascular anatomic alterations in individuals who have pedigree for SH showed inconsistent results [5–6]. These discrepancies are probably due to selection criteria adopted with respect to the age and body weight of the subjects. On the other hand, it has been suggested that alterations in left ventricular mass and or function may precede development of sustained elevation in blood pressure; and may be genetically determined [7]. In the Tecumseh Offspring Study [8] heredity was found to account for a small but definite proportion of variations of LVM and the predictive power of heredity of LVM was only second to that of the child’s height. Similarly, in the Framingham Heart Study [9], heredity was also found to explained small but discernible proportion of variations in LVM. The aim of the present study was to evaluate left ventricular structure in OHP to determine whether subclinical cardiovascular dysfunction exists before the development of overt hypertension.

## Methods

A simple random sample (using random numbers) of patients who were hypertensive and attending Cardiovascular Clinic of the University of Ilorin Teaching Hospital, Ilorin was conducted. The selected patients were requested to bring one of their eligible children between the ages of 15–25 years for assessment (OHP group). The same procedure was employed to select age and sex-matched children without parental hypertension (ONP) from Medical Outpatient Department to serve as controls. Ethical approval was obtained from the Ethics and Research Committee of the hospital and informed consent was obtained from each participant.

All individuals who were pregnant, on cardio active drugs, had cardio-pulmonary disease; and those with organic heart murmur and renal bruit were not eligible to participate. Similarly, those with structural heart disease and poor echocardiographic windows were excluded from the study. A thorough history to exclude cardiovascular disease in the participants was taken and anthropometric parameters, blood pressure and other relevant clinical examination were performed on all subjects. Blood pressure was measured at the left arm in a comfortable position after about 10 minutes rest using an appropriate cuff. However, if the blood pressure was equal or greater than 10mmHg at the right arm, the latter blood pressure was used as the blood pressure of the subjects. An average of three measurements was used as the blood pressure. Individuals with blood pressure ≥ 140/90 mmHg or on anti-hypertensive drugs were also excluded. Electrocardiogram was done in standard position and subsequently echocardiogram. A long parasternal 2D guided M-mode echocardiogram was done and measurement taken according to the recommendations of the American Society of Echocardiography (ASE) (Schiller et al, 1989) [10]. Parameters recorded included: LV internal dimension in diastole (LVIDd), LV internal dimension in systole (LVIDs), interventricular septal thickness in diastole (IVSd), interventricular septal thickness in systole (IVSs), posterior wall thickness in diastole (PWd), posterior wall thickness in systole (PWs), LV ejection fraction (EF) and fractional shortening (FS). Others include right ventricular dimension in diastole (RVd), aortic dimension (AOD) and left atrial dimension (LAD). LV mass (LVM) was calculated using the formula [11]: 0.8×(1.04 ×((LVIDd+IVSd+PWd)^3^−(LVIDd)^3^))+0.6. Left ventricular mass index (LVMI) was determined using formula: LVM/BSA, where BSA is the body surface area.Relative wall thickness (RWT) was calculated by the formula [12]: 2×PWd/LVIDd. The pattern of LV remodelling was determined using LVMI and RWT. Increased RWT was present if RWT was ≥0.4512. LV geometric pattern was classified using RWT and LVMI as follows: Normal geometry=normal LVMI and RWT; Concentric remodelling= normal LVMI and RWT ≥0.45; Eccentric LV hypertrophy= increased LVMI and RWT <0.45; Concentric LV hypertrophy= increased LVMI and RWT ≥0.45

### Statiscal analysis

Statistical analysis was performed using the SPSS Version 15 and numerical values were presented as mean±standard deviation. Student t-test was used to compare means of continuous variables while chi-square test was used to compare means of proportions. Test of correlation was done using the Pearson's correlation method. Stepwise regression analysis using 0.05 as entry probability and 0.10 as removal probability was used to determine predictors of LVM. A statistically significant association was taken at P

## Results

**Figure 1:**
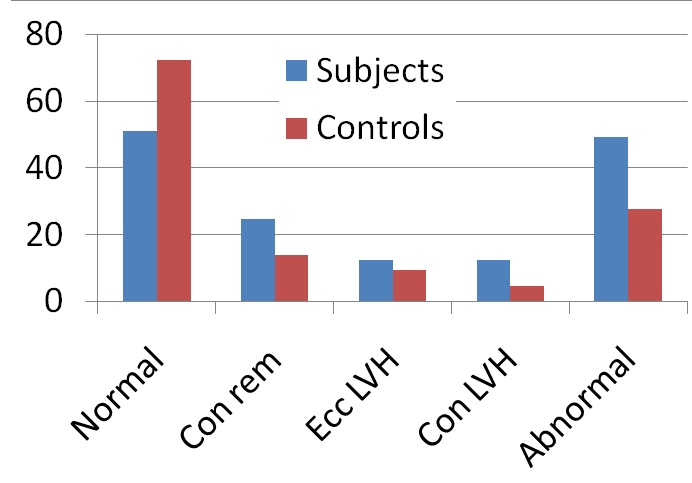
Left ventricular geometric patterns in the subjects and controls. Normal: Normal-geometry; Con rem: Concentric remodeling; Ecc LVH: Eccentric Left Ventricular Hypertrophy; Con LVH: Concentric left Ventricular Hypertrophy; Abnormal: Abnormal geometry

The demographic characteristics of study participants are presented in Table 1. Sixty-five normotensive OHP with 65-age and sex-matched normotensive ONP were studied. They consisted of 18 (27.7%) females and 47 (72.3%) males in each group and their ages ranged from 15 to 25 years. The mean age of OHP was similar to that of ONP group (p>0.05). The anthropometric parameters showed that the body mass index, waist and hip circumference were similar in the two groups. Twenty-two (33.8%) subjects had history of hypertension in their fathers, 29 (44.6%) in mothers and 14 (21.5%) in both parents. The mean systolic and diastolic blood pressures though higher in OHP than ONP group, the difference did not reach statistical significance. Parameters of right ventricular and left ventricular dimensions are showed in Table 2. The right ventricular dimension in diastole, LVIDd and LVIDs; LAD and AOD were similar between the subjects and controls. However, the mean PWd in OHP was higher (p<0.001) than that of ONP group. Similarly, LVM, LVMI and RWT were significantly increased in the subjects than in controls (p<0.01, <0.01 and 0.001 respectively). On the other hand, parameters of left ventricular systolic function (ejection fraction and fractional shortening) were similar between the two groups. Left ventricular geometric patterns are shown in Figure 1. The incidence of abnormal left ventricular geometric patterns was significantly higher (p<0.01) in subjects than controls. In the subjects, SBP, DBP, waist and hip circumference as well as ECG voltage criteria correlated positively with LVMI while negative correlation was observed between LVMI and left ventricular ejection fraction (Table 3). Only waist circumference was independently associated with LVMI (Mean square=3724.339, F=20.745, P<0.001) in the patients.

**Table 1:** Clinical characteristics of the study group

Parameter	Subjects	Control	P-value
		
	Mean (SD)	Mean (SD)	
Number	65	65	
**Sex**			
Male	47	47	
Female	18	18	
Age (years)	21.6 (0.29)	21.7 (0.26)	0.88
Body mass index (kg/m^2^)	22.1 (3.0)	21.7 (2.8)	0.405
WC (cm)	80.4 (8.9)	78.3 (7.1)	0.215
HC (cm)	89.5 (9.1)	88.8 (6.8)	0.653
SBP (mmHg)	118.2 (8.99)	115.9 (8.76)	0.138
DBP (mmHg)	75.6 (7.6)	72.8 (8.8)	0.06

WC-waist circumference, HC-hip circumference, SBP-systolic blood pressure, DBP-diastolic blood pressure

* statistically significant

**Table 2:** Echocardiographic parameters of the subjects and controls

Dimension	Subjects	Controls	p-value
		
	Mean (SD)	Mean (SD)	
RVd (mm)	13.3 (4.5)	14.3 (3.6)	0.14
IVSd (mm)	11.2 (2.4)	11.2 (2.7)	0.97
PWd (mm)	9.7 (2.3)	8.5 (1.8)	0.001*
LVDd (mm)	45.6 (5.3)	45.4 (5.8)	0.8
LVDs (mm)	29.2 (6.5)	29.1 (4.8)	0.92
Ejection fraction (%)	66.8 (8.4)	65.5 (8.8)	0.4
Fractional shortening (%)	37.1 (6.6)	36.2 (7.2)	0.5
LVM (g)	196.9 (63.8)	175.2 (58.8)	0.01*
LVMI (g/m^2^)	114.5 (32.6)	103.2 (26.1)	0.01*
AOD (mm)	27.3 (2.9)	27.2 (3.0)	0.78
LAD (mm)	30.7 (4.5)	30.8 (5.0)	0.88
RWT	0.43 (0.11)	0.38 (0.09)	0.001*

Key: RVd-right ventricular dimension, IVSd-interventricular septum in diastole, PWd- posterior wall in diastole, LVDd-left ventricular dimension in diastole, LVDs-left ventricular dimension in systole, LVM-left ventricular mass, LVMI-left ventricular mass index, AOD-aortic dimension, LAD-left atrial dimension, RWT-relative wall thickness;

* statistically significant

**Table 3:** Correlates of Left ventricular mass index in normotensive offspring of hypertensive parents

Parameters	Pearson Correlation with LVMI	P-value
Age	0.02	0.9
BMI	0.159	0.21
WC	0.418	0.01*
HC	0.326	0.01*
QTc	0.18	0.15
ECG Voltage (SV2+RV6)	0.47	0.01*
LV ejection fraction	−0.255	0.01*

BMI-body mass index, WC-waist circumference, HC-hip circumference, ECG-electrocardiogram, QTc-corrected QT interval, LVMI-left ventricular mass Index;

* statistically significant

## Discussion

The main purpose of the present study was to evaluate left ventricular structure in normotensive OHP with the aim of detecting early abnormalities (if any) preceding onset of SH. The results showed that the LVMI as well as PWd were significantly higher in OHP than ONP (p<0.001 and 0.01 respectively). Similarly, prevalence of abnormal left ventricular geometric pattern was higher in the OHP compared with ONP (Figure 1). These results further confirm the findings of previous studies which demonstrated increased LVM in normotensive OHP against ONP [13–14]. On the other hand, our results contrast that of Jalal et al [15] and Graetinger et al [16] who did not find significant difference in LVM between the two groups. However, it is significant to note that our patients were older than those evaluated by Jalal et al [15] but similar in age to offspring of Dutch Hypertension and Offspring Study [17]. Therefore, age does appear to be an important factor in the expression of LVM phenotypes in OHP and; it is possible that difference in LVM and left ventricular structure may not be apparent at younger age group (<18 years) as alluded to by Jalal et al. Although, the clinical impact of left ventricular hypertrophy on morbidity and mortality is well known in cardiovascular diseases in adults, nevertheless, the origin of increased LVM and its clinical significance is still debated in OHP. Some investigators have suggested that increased LVM may be a forerunner of hypertension and future cardiovascular events in these individuals [18–19]. The association of obesity with LVM in normotensive children has been demonstrated in some studies [20]. Our study showed that the waist circumference correlated positively with LVMI; and at the same time an independent predictor of LVMI. Waist circumference reflects the visceral distribution of fat and may be more important than the body mass index in this group of individuals [21].

Published observations had also shown mean 24-hour ambulatory systolic and diastolic blood pressure values to be an important determinant of LVM in hypertensive children and OHP [22–23]. In our study, both mean systolic and diastolic blood pressures were similar in the two groups. On the other hand, the role of genetic contribution to the variations in LVM has been recognized and these variations are thought to be co-inherited with risk of hypertension in genetically predisposed individual [24]. However, the selection of our subjects was based on parental and offspring's blood pressures but not on parental LVM. Therefore, the influence of parental LVM on variations of LVM in the offspring was not demonstrated in the present study.

Assessment of left ventricular geometry further refined risk associated with LVH in individuals with systemic hypertension [25]. This study showed higher incidence of abnormal left ventricular geometric patterns in the subjects compared with the controls. The presence of abnormal left ventricular geometry in normotensive offspring of hypertensive parents suggests that alterations in left ventricular structure may also have genetic basis. This is supported by a recent study by Lam et al [26] which showed familiar aggregation of left ventricular geometry in a two generation community-based sample.

The results of the present study also showed that the parameters of left ventricular systolic function at rest were similar between the OHP and ONP groups. This is not surprising because prior investigations have shown that even in hypertensive individuals, alterations in the indices of left ventricular systolic function occur late in the course of the disease [27].

## Conclusion

In conclusion, normotensive offspring of hypertensive parents have alterations in left ventricular mass and geometry even before they have sustained elevation of blood pressure. OHP should be considered as a special group which needs early dietary adjustment and lifestyles modification in order to prevent future cardiovascular events.
